# Pharmacokinetic-Pharmacodynamic Target Attainment Analyses as Support for Meropenem-Vaborbactam Dosing Regimens and Susceptibility Breakpoints

**DOI:** 10.1128/aac.02130-21

**Published:** 2022-11-14

**Authors:** S. M. Bhavnani, M. Trang, D. C. Griffith, O. Lomovskaya, J. P. Hammel, J. S. Loutit, S. K. Cammarata, M. N. Dudley, P. G. Ambrose, C. M. Rubino

**Affiliations:** a Institute for Clinical Pharmacodynamics, Inc., Schenectady, New York, USA; b Rempex Pharmaceuticals, San Diego, California, USA; c Melinta Therapeutics, Inc., Lincolnshire, Illinois, USA

**Keywords:** meropenem-vaborbactam, pharmacokinetic-pharmacodynamic target attainment, dose selection, susceptibility breakpoints

## Abstract

Meropenem-vaborbactam is a fixed-dose beta-lactam/beta-lactamase inhibitor with potent *in vitro* and *in vivo* activity against Klebsiella pneumoniae carbapenemase (KPC)-producing *Enterobacterales*. Pharmacokinetic-pharmacodynamic (PK-PD) target attainment analyses were undertaken using population pharmacokinetic models, nonclinical PK-PD targets for efficacy, *in vitro* surveillance data, and simulation to provide support for 2 g meropenem-2 g vaborbactam every 8 h (q8h) administered as a 3-h intravenous (i.v.) infusion, and dosing regimens adjusted for patients with renal impairment. Simulated patients varying by renal function measure (estimated glomerular filtration rate [eGFR], mL/min/1.73 m^2^ and absolute eGFR, mL/min) and resembling the clinical trial population (complicated urinary tract infection, including acute pyelonephritis) were generated. The PK-PD targets for meropenem, the percentage of time on day 1 that free-drug plasma concentrations were above the MIC (%T>MIC), and vaborbactam, the ratio of free-drug plasma area under the concentration-time curve (AUC) on day 1 to the MIC (AUC:MIC ratio), were calculated. Percent probabilities of achieving meropenem free-drug plasma %T>MIC and vaborbactam free-drug plasma AUC:MIC ratio targets were assessed. MIC distributions for *Enterobacterales*, KPC-producing *Enterobacterales*, and Pseudomonas aeruginosa were considered as part of an algorithm to assess PK-PD target attainment. For assessments of free-drug plasma PK-PD targets associated with a 1-log_10_ CFU reduction from baseline, percent probabilities of PK-PD target attainment ranged from 81.3 to 100% at meropenem-vaborbactam MIC values of 4 or 8 μg/mL among simulated patients. The results of these PK-PD target attainment analyses provide support for a dosing regimen of 2 g meropenem-2 g vaborbactam q8h administered as a 3-h i.v. infusion, with dosing regimens adjusted for patients with renal impairment and a meropenem-vaborbactam susceptibility breakpoint of ≤8 μg/mL (tested with a fixed vaborbactam concentration of 8 μg/mL) for *Enterobacterales* and P. aeruginosa based on these dosing regimens.

## INTRODUCTION

Meropenem-vaborbactam is a fixed-dose beta-lactam/beta-lactamase inhibitor combination of meropenem, a carbapenem, and vaborbactam, a cyclic boronic acid β-lactamase inhibitor with potent *in vitro* and *in vivo* activity against Klebsiella pneumoniae carbapenemase (KPC)-producing *Enterobacterales* (1–4). Approval of meropenem-vaborbactam as a fixed dosing regimen of 2 g/2 g administered every 8 h (q8h) as a 3-h intravenous (i.v.) infusion for the treatment of patients with complicated urinary tract infections (cUTI), including acute pyelonephritis (AP), was granted by the U.S. Food and Drug Administration (FDA) in 2017 (3). In addition to cUTI or AP, this dosing regimen was also approved for the treatment of patients with hospital-acquired pneumonia, including ventilator-associated pneumonia, and those with complicated intra-abdominal infections by the European Medicines Agency (EMA) in 2018 (4). As described below, data from pharmacometric analyses for meropenem-vaborbactam represented an important element of the new drug application (NDA) submitted to the U.S. FDA and the marketing authorization application (MAA) submitted to the EMA.

The current drug development paradigm for antimicrobial agents includes the use of population pharmacokinetic (PK) models together with nonclinical pharmacokinetic-pharmacodynamic (PK-PD) targets for efficacy, *in vitro* surveillance data, and simulation to carry out PK-PD target attainment analyses. Results of such analyses are used to provide dose decision support in both early- and late-stage development. When conducted in late-stage development, PK data from the target patient populations allow for confirmation of decisions made in early-stage development. Additionally, PK data from special populations, such as subjects with renal impairment, can be used to inform dosing recommendations for such patients. This strategy is useful for programs for which there are limited or no clinical trial data. Lastly, such data are also used to provide recommendations for interpretive criteria for the *in vitro* susceptibility testing for antibacterial agents against a given pathogen (5).

Population PK models for meropenem and vaborbactam were developed using PK data from two phase 1 studies, one which evaluated subjects after administration of single and multiple doses (6), and one which evaluated subjects with renal impairment (7), and two phase 3 studies, TANGO I (study 505; ClinicalTrials.gov registration no. NCT02166476 [8]) and TANGO II (study 506; ClinicalTrials.gov registration no. NCT02168946 [9]). For the population PK models included in the NDA submitted to the U.S. FDA, the data set included all available data from the TANGO II study, which was ongoing at the time. After the completion of the TANGO II study, data from an additional 27 patients were used to refine the population PK models, which were used to support the MAA submitted to the EMA (10).

The use of each set of the above-described population PK models (10), together with nonclinical PK-PD data (2, 11–13), *in vitro* surveillance data (1, 14–16), and simulation, allowed for the conduct of PK-PD target attainment analyses for meropenem-vaborbactam for each region. As described herein, results of such analyses were used to provide support for 2 g meropenem-2 g vaborbactam q8h administered as a 3-h i.v. infusion and dose adjustments for patients with renal impairment based on different measures of renal function that were approved by the U.S. FDA and the EMA. Such data were also useful to provide support for interpretive criteria for the *in vitro* susceptibility testing of meropenem-vaborbactam against *Enterobacterales* and Pseudomonas aeruginosa, which were considered by the various antimicrobial standard development organizations (17–19).

## RESULTS

### Summary of meropenem and vaborbactam free-drug plasma exposures.

Meropenem-vaborbactam dosing regimens by renal function group that best matched day 1 and steady-state meropenem and vaborbactam free-drug plasma area under the concentration-time curve (AUC) from 0 to 24 h (AUC_0–24_) values for simulated patients with cUTI or AP and normal renal function are shown in Table 1. Measures of renal function were estimated glomerular filtration rate (eGFR; mL/min/1.73 m^2^) and absolute eGFR (mL/min) for the U.S. FDA and EMA submissions, respectively. Summary statistics for day 1 and steady-state free-drug plasma AUC_0–24_ values for both agents among simulated patients with cUTI or AP by renal function group for both the U.S. FDA and EMA submissions are provided in Table S1 in the supplemental material. As expected, there was substantial overlap among the distributions of day 1 and steady-state free-drug plasma AUC_0–24_ values across renal function groups for both meropenem and vaborbactam. Distributions of steady-state vaborbactam free-drug plasma AUC_0–24_ values demonstrated increased AUC_0–24_ values for lower eGFR or absolute eGFR groups. Using the final population PK model for vaborbactam (10), *post hoc* steady-state free-drug plasma AUC_0–24_ values in patients with such low eGFR or absolute eGFR values from the TANGO II study were examined. Trends similar to those described above for simulated patients with lower eGFR or absolute eGFR groups were observed for the patients with renal impairment in the TANGO II study but without any corresponding safety events (data not shown). Table S2 shows the summary statistics for day 1 and steady state free-drug plasma AUC_0–24_ values for both agents among 3,245 simulated patients with cUTI or AP and various levels of renal function (i.e., resembling the clinical trial population) for each submission. Simulated patients in this population also received meropenem-vaborbactam dosing regimens by renal function group as described in Table 1. Distributions of free-drug plasma AUC_0–24_ values for each drug were similar between the two submission packages.

**TABLE 1:** Meropenem-vaborbactam dosing regimens administered to simulated patients by renal function group

Meropenem-vaborbactam dosing regimen (infused over 3 hours)	Region of regulatory submission, renal function measure	
	U.S. FDA submission, eGFR (mL/min/1.73 m^2^)	EMA submission, absolute eGFR (mL/min)
2 g meropenem and 2 g vaborbactam q8h	≥150 to ≤200	≥150 to ≤200
2 g meropenem and 2 g vaborbactam q8h	≥50 to <150	≥40 to <150
1 g meropenem and 1 g vaborbactam q8h	≥30 to <50	≥20 to <40
1 g meropenem and 1 g vaborbactam q12h	≥15 to <30	≥10 to <20
0.5 g meropenem and 0.5 g vaborbactam q12h	≥0 to <15	≥0 to <10

### Evaluation of PK-PD target attainment results.

Table 2 and Table 3 show the percent probabilities of PK-PD target attainment by MIC and overall on day 1 among simulated patients with cUTI or AP by renal function group after administration of meropenem-vaborbactam dosing regimens evaluated for the U.S. FDA and EMA submissions, respectively, as described in Table 1. For each set of results, percent probabilities of PK-PD target attainment are shown by the percentage of time on day 1 that meropenem free-drug plasma concentrations were above the MIC (%T>MIC) ≥30, 35, 40, and 45% targets. As described in Materials and Methods, each meropenem free-drug plasma %T>MIC target was evaluated in combination with the ratio of the day 1 vaborbactam free-drug plasma AUC to MIC (AUC:MIC ratio) target of 38, which is associated with a 1-log_10_ CFU reduction from baseline. The results of the PK-PD target attainment analyses are presented by the meropenem-vaborbactam MIC distribution assessed. The three MIC distributions assessed were based on collections of *Enterobacterales*, KPC-producing *Enterobacterales*, and P. aeruginosa isolates.

**TABLE 2:** Percent probabilities of PK-PD target attainment by meropenem-vaborbactam MIC and overall on day 1 based on the assessment of four meropenem free-drug plasma %T>MIC targets and MIC data for *Enterobacterales*, KPC-producing *Enterobacterales*, and P. aeruginosa isolates among simulated patients with cUTI or AP by eGFR group after administration of meropenem-vaborbactam dosing regimens for the U.S. FDA submission^*a*^

Pathogen^*b*^	MVB MIC (μg/mL)	Percent probabilities of PK-PD target attainment by MVB MIC and overall for meropenem free-drug plasma %T>MIC targets^*c*^ assessed among simulated patients with cUTI or AP by eGFR (mL/min/1.73 m^2^) group																			
		Meropenem free-drug plasma %T>MIC ≥30					Meropenem free-drug plasma %T>MIC ≥35					Meropenem free-drug plasma %T>MIC ≥40					Meropenem free-drug plasma %T>MIC ≥45				
		≥0 to <15	≥15 to <30	≥30 to <50	≥50 to <150	≥150 to ≤200	≥0 to <15	≥15 to <30	≥30 to <50	≥50 to <150	≥150 to ≤200	≥0 to <15	≥15 to <30	≥30 to <50	≥50 to <150	≥150 to ≤200	≥0 to <15	≥15 to <30	≥30 to <50	≥50 to <150	≥150 to ≤200
All ENT	2	100	100	100	100	100	100	100	100	100	100	100	100	100	100	100	100	100	100	100	99.9
	4	99.9	100	100	100	100	99.6	100	100	100	100	99.6	99.9	100	100	99.9	99.5	99.8	99.5	99.8	99.8
	8	97.3	99.6	97.9	100	99.9	96.7	99.2	96.3	99.7	99.7	95.9	97.8	92.9	99.0	98.7	93.8	96.6	88.4	96.8	95.3
	16	74.5	89.7	75.0	95.2	93.5	71.2	86.0	68.6	91.8	88.2	66.5	81.6	60.8	86.0	81.5	62.0	76.1	52.8	76.0	71.7
	Overall^*d*^	99.6	99.7	99.6	99.7	99.7	99.5	99.6	99.5	99.7	99.7	99.5	99.6	99.5	99.6	99.6	99.5	99.6	99.5	99.6	99.6
KPC-ENT	2	100	100	100	100	100	100	100	100	100	100	100	100	100	100	100	100	100	100	100	99.9
	4	99.9	100	99.7	99.9	99.8	99.6	100	99.7	99.9	99.6	99.6	99.9	99.7	99.9	99.4	99.5	99.8	99.2	99.7	99.1
	8	88.5	97.8	85.7	93.7	90.6	88.0	97.4	84.3	92.2	89.0	87.2	96.0	81.3	90.8	86.6	85.4	94.8	77.5	88.2	82.0
	16	20.2	49.0	18.3	49.2	33.4	18.6	45.3	14.9	42.5	27.0	16.7	42.7	11.4	35.6	21.5	15.1	39.0	8.80	28.3	15.4
	Overall^*d*^	99.5	99.6	99.5	99.6	99.6	99.5	99.6	99.5	99.6	99.5	99.5	99.6	99.5	99.6	99.5	99.5	99.6	99.4	99.5	99.5
PSA	2	100	100	100	100	100	100	100	100	100	100	100	100	100	100	100	100	100	100	100	99.9
	4	100	100	100	100	100	99.8	100	100	100	100	99.8	99.9	100	100	99.9	99.7	99.8	99.5	99.8	99.8
	8	97.7	99.7	98.2	100	99.9	97.2	99.4	96.8	99.8	99.8	96.6	98.0	94.1	99.3	98.9	94.9	96.8	90.3	97.3	95.9
	16	76.6	90.6	78.6	96.0	94.7	73.3	86.8	71.5	92.7	90.3	69.1	82.9	63.4	86.7	84.4	64.9	77.9	55.8	77.7	75.2
	Overall^*d*^	92.2	93.9	92.4	95.0	94.6	91.8	93.4	91.7	94.4	94.1	91.4	92.9	90.8	93.6	93.3	90.9	92.4	89.9	92.5	92.1

a Light gray shaded cells indicate percent probabilities of PK-PD target attainment by MIC ≥90%.

b ENT, *Enterobacterales*; KPC-ENT, KPC-producing *Enterobacterales*; MVB, meropenem-vaborbactam; PSA, P. aeruginosa.

c Based on the assessment of a vaborbactam free-drug plasma AUC:MIC ratio target of 38, which was associated with a 1-log_10_ CFU reduction from baseline in a neutropenic murine thigh-infection model.

d Represents the weighted percent probability of PK-PD target attainment over the meropenem-vaborbactam MIC distribution for a given pathogen.

**TABLE 3:** Percent probabilities of PK-PD target attainment by meropenem-vaborbactam MIC and overall on day 1 based on the assessment of four meropenem free-drug plasma %T>MIC targets and MIC data for *Enterobacterales*, KPC-producing *Enterobacterales*, and P. aeruginosa isolates among simulated patients with cUTI or AP by absolute eGFR group after administration of meropenem-vaborbactam dosing regimens for the EMA submission^*a*^

Pathogen^*b*^	MVB MIC (μg/mL)	Percent probabilities of PK-PD target attainment by MVB MIC and overall for meropenem free-drug plasma %T>MIC targets^*c*^ assessed among simulated patients with cUTI or AP by absolute eGFR (mL/min) group																			
		Meropenem free-drug plasma %T>MIC ≥ 30%					Meropenem free-drug plasma %T>MIC ≥ 35%					Meropenem free-drug plasma %T>MIC ≥ 40%					Meropenem free-drug plasma %T>MIC ≥ 45%				
		≥0 to <10	≥10 to <20	≥20 to <40	≥40 to <150	≥150 to ≤200	≥0 to <10	≥10 to <20	≥20 to <40	≥40 to <150	≥150 to ≤200	≥0 to <10	≥10 to <20	≥20 to <40	≥40 to <150	≥150 to ≤200	≥0 to <10	≥10 to <20	≥20 to <40	≥40 to <150	≥150 to ≤200
All ENT	2	100	100	100	100	100	100	100	100	100	100	100	100	100	100	100	100	100	100	100	100
	4	99.7	100	100	99.9	100	99.7	100	100	99.9	100	99.6	100	100	99.9	100	99.6	100	99.9	99.8	99.8
	8	96.9	99.9	99.7	99.8	99.6	96.4	99.9	99.2	99.2	99.5	96.0	99.8	98.6	98.8	97.9	94.9	99.7	97.3	97.0	93.8
	16	73.2	95.5	91.4	95.4	92.3	70.4	94.4	89.2	92.3	88.2	67.9	92.5	85.6	88.0	82.2	64.4	90.0	80.8	80.9	72.6
	Overall^*d*^	99.6	99.7	99.7	99.7	99.7	99.5	99.7	99.7	99.7	99.7	99.5	99.7	99.6	99.7	99.6	99.5	99.7	99.6	99.6	99.6
KPC-ENT	2	100	100	100	100	100	100	100	100	100	100	100	100	100	100	100	100	100	100	100	100
	4	99.7	100	100	99.9	99.2	99.7	100	100	99.9	99.2	99.6	100	100	99.9	99.2	99.6	100	99.9	99.8	99.0
	8	91.5	99.7	97.3	92.3	86.3	91.0	99.7	96.8	91.8	86.1	90.8	99.7	96.2	91.1	84.6	89.7	99.6	95.1	89.9	81.2
	16	28.5	81.5	60.7	53.8	33.6	27.4	79.7	59.1	50.6	29.6	26.2	78.0	57.2	47.0	25.4	24.9	75.6	54.7	42.2	21.8
	Overall^*d*^	99.5	99.7	99.7	99.6	99.5	99.5	99.7	99.6	99.6	99.5	99.5	99.7	99.6	99.6	99.5	99.5	99.7	99.6	99.6	99.5
PSA	2	100	100	100	100	100	100	100	100	100	100	100	100	100	100	100	100	100	100	100	100
	4	99.8	100	100	100	100	99.8	100	100	100	100	99.7	100	100	100	100	99.7	100	99.9	99.9	99.8
	8	97.8	99.9	99.8	99.9	99.8	97.3	99.9	99.3	99.5	99.8	97.0	99.9	98.8	99.2	98.6	96.1	99.8	97.8	97.7	95.4
	16	75.9	96.0	92.0	96.4	94.0	73.4	94.7	89.6	94.3	90.2	70.7	92.9	85.9	89.9	83.4	67.2	90.7	81.4	82.8	74.6
	Overall^*d*^	92.2	95.0	94.4	95.2	94.7	91.9	94.6	94.0	94.7	94.0	91.6	94.3	93.5	94.0	93.1	91.2	94.0	93.0	93.0	92.0

a Light gray shaded cells indicate percent probabilities of PK-PD target attainment by MIC ≥90%.

b ENT, *Enterobacterales*; KPC-ENT, KPC-producing *Enterobacterales*; MVB, meropenem-vaborbactam; PSA, P. aeruginosa.

c Based on the assessment of a vaborbactam free-drug plasma AUC:MIC ratio target of 38, which was associated with a 1-log_10_ CFU reduction from baseline in a neutropenic murine-thigh-infection model.

d Represents the weighted percent probability of PK-PD target attainment over the meropenem-vaborbactam MIC distribution for a given pathogen.

**FIG 1:**
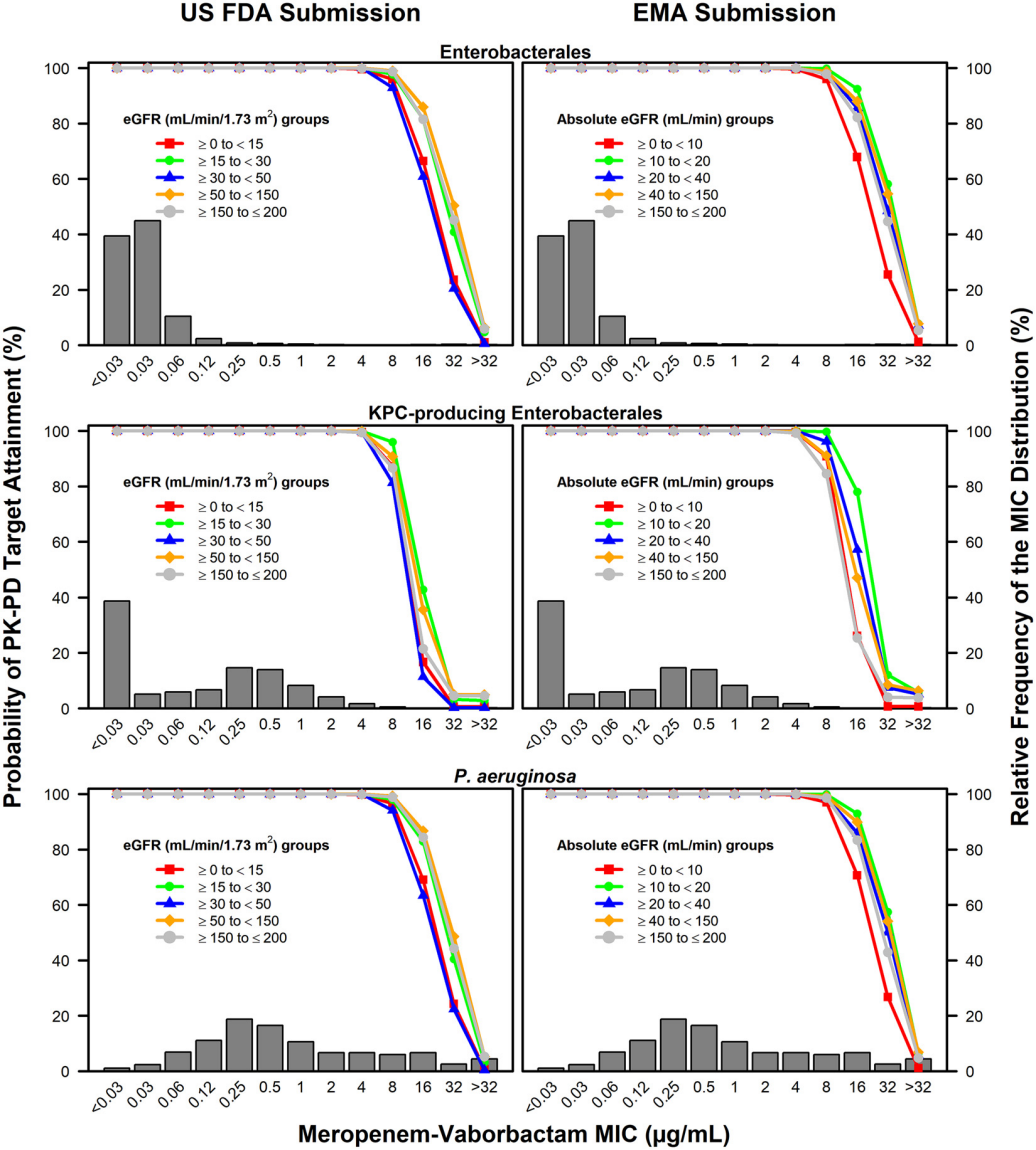
Percent probabilities of PK-PD target attainment by meropenem-vaborbactam MIC on day 1 based on the assessment of the meropenem free-drug plasma %T>MIC ≥40% target and MIC data for collections of isolates among simulated patients with cUTI or AP by eGFR and absolute eGFR groups after administration of meropenem-vaborbactam dosing regimens for the U.S. FDA and EMA submissions, overlaid on the meropenem-vaborbactam MIC distributions for *Enterobacterales* (top), KPC-producing *Enterobacterales* (middle), and P. aeruginosa (bottom) isolates.

The above-described percent probabilities of PK-PD target attainment by MIC based on the meropenem free-drug plasma %T>MIC ≥40% target (i.e., which is associated with a 1-log_10_ CFU reduction from baseline) among simulated patients by eGFR and absolute eGFR groups after administration of meropenem-vaborbactam dosing regimens for both the U.S. FDA and EMA submissions are shown overlaid on the meropenem-vaborbactam MIC distributions for *Enterobacterales*, KPC-producing *Enterobacterales*, and P. aeruginosa isolates, in the top, middle, and bottom panels, respectively, of Fig. 1. Such data are shown based on the assessment of meropenem free-drug plasma %T>MIC ≥30, 35, and 45% targets in Fig. S1, S2, and S3, respectively.

**FIG 2:**
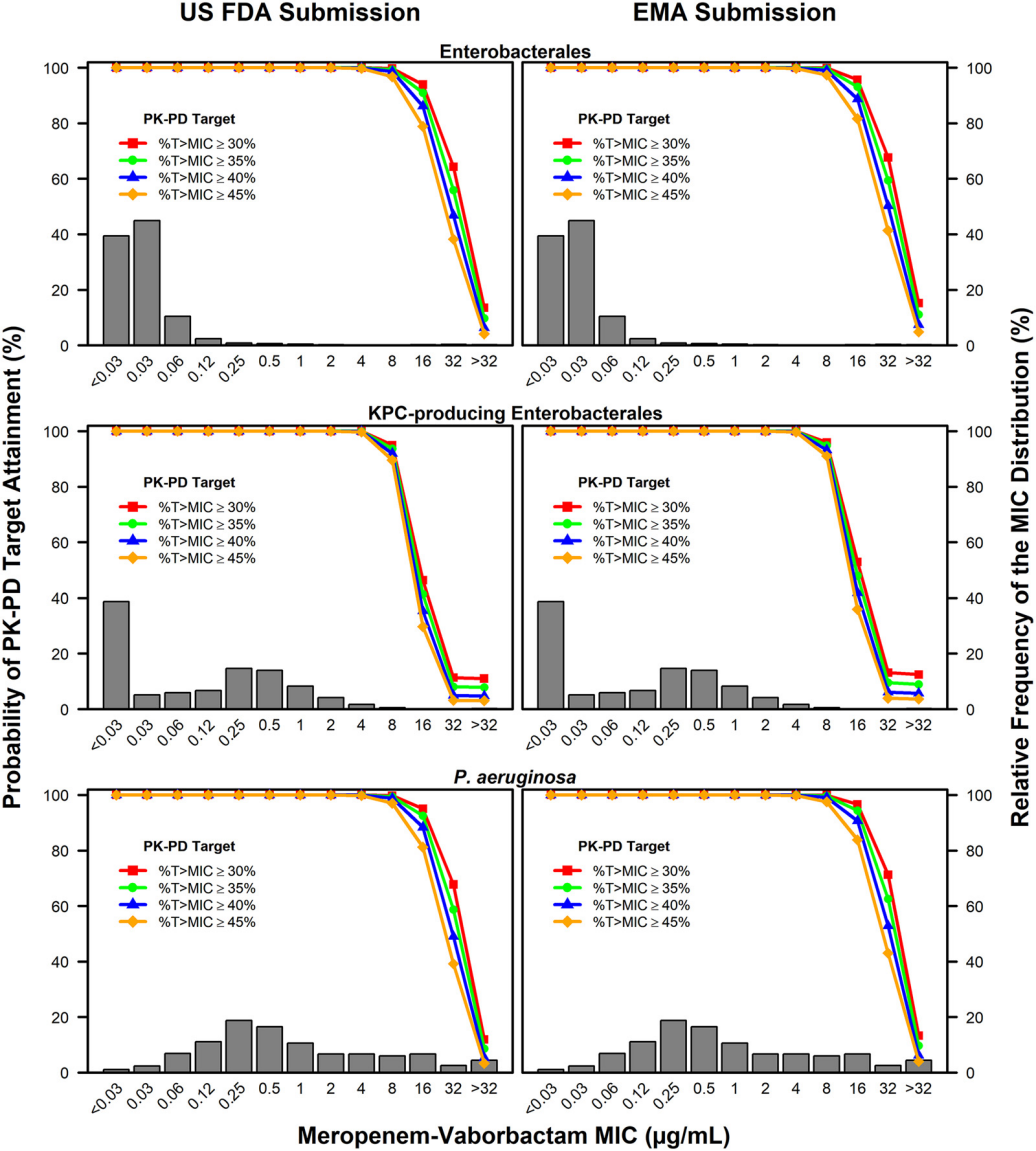
Percent probabilities of PK-PD target attainment by meropenem-vaborbactam MIC on day 1 based on the assessment of four meropenem free-drug plasma %T>MIC targets and MIC data for collections of isolates among simulated patients with cUTI or AP and various levels of renal function after administration of meropenem-vaborbactam dosing regimens for the U.S. FDA and EMA submissions, overlaid on the meropenem-vaborbactam MIC distributions for *Enterobacterales* (top), KPC-producing *Enterobacterales* (middle), and P. aeruginosa (bottom) isolates.

Table 4 shows the percent probabilities of PK-PD target attainment by MIC and overall on day 1 based on the assessment of the four above-described meropenem free-drug plasma %T>MIC targets and MIC distributions for *Enterobacterales*, KPC-producing *Enterobacterales*, and P. aeruginosa isolates among simulated patients with cUTI or AP and various levels of renal function after administration of meropenem-vaborbactam dosing regimens for the U.S. FDA and EMA submissions. As with the above-described assessment of simulated patients by renal function group, each meropenem free-drug plasma %T>MIC target was evaluated in combination with the vaborbactam free-drug plasma AUC:MIC ratio target of 38, which is associated with a 1-log_10_ CFU reduction from baseline. These percent probabilities of PK-PD target attainment by MIC for the U.S. FDA and EMA submissions are shown overlaid on the above-described meropenem-vaborbactam MIC distributions in Fig. 2.

**TABLE 4:** Percent probabilities of PK-PD target attainment by meropenem-vaborbactam MIC and overall on day 1 based on the assessment of four meropenem free-drug plasma %T>MIC targets and MIC data for *Enterobacterales*, KPC-producing *Enterobacterales*, and P. aeruginosa isolates among simulated patients with cUTI or AP and various levels of renal function after administration of meropenem-vaborbactam dosing regimens for the U.S. FDA and EMA submissions^*a*^

Pathogen^*b*^	MVB MIC (μg/mL)	Percent probabilities of PK-PD target attainment by MVB MIC and overall for meropenem free-drug plasma %T>MIC targets^*c*^ assessed among simulated patients with cUTI or AP and various levels of renal function by region of submission							
		U.S. FDA				EMA submission			
		%T>MIC ≥ 30%	%T>MIC ≥ 35%	%T>MIC ≥ 40%	%T>MIC ≥ 45%	%T>MIC ≥ 30%	%T>MIC ≥ 35%	%T>MIC ≥ 40%	%T>MIC ≥ 45%
*Enterobacterales*	2	100	100	100	100	100	100	100	100
	4	100	100	99.9	99.8	100	100	99.9	99.8
	8	99.7	99.3	98.6	96.7	99.9	99.7	99.0	97.4
	16	93.9	90.9	86.2	78.9	95.7	93.1	88.7	81.7
	32	64.3	55.9	46.8	38.2	67.7	59.4	50.3	41.4
	Overall^*d*^	99.7	99.7	99.6	99.6	99.7	99.7	99.7	99.6
KPC-producing *Enterobacterales*	2	100	100	100	100	100	100	100	100
	4	100	100	99.9	99.7	100	100	99.9	99.7
	8	94.9	93.7	92.0	89.6	95.9	94.9	93.2	91.0
	16	46.3	41.4	35.4	29.7	52.9	48.0	41.8	36.0
	32	11.3	8.01	4.90	3.14	13.2	9.49	6.10	3.94
	Overall^*d*^	99.6	99.6	99.6	99.5	99.6	99.6	99.6	99.6
P. aeruginosa	2	100	100	100	100	100	100	100	100
	4	100	100	100	99.8	100	100	100	99.8
	8	99.8	99.4	98.8	97.0	99.9	99.7	99.1	97.6
	16	95.1	92.5	88.4	81.2	96.6	94.4	90.6	83.9
	32	67.8	58.7	49.0	39.2	71.3	62.6	52.8	43.1
	Overall^*d*^	94.9	94.4	93.6	92.7	95.2	94.6	94.0	93.0

a Light gray shaded cells indicate percent probabilities of PK-PD target attainment by MIC ≥90%.

b ENT, *Enterobacterales*; KPC-ENT, KPC-producing *Enterobacterales*; MVB, meropenem-vaborbactam; PSA, P. aeruginosa.

c Based also on the assessment of a vaborbactam free-drug plasma AUC:MIC ratio target of 38, which was associated with a 1-log_10_ CFU reduction from baseline in a neutropenic murine-thigh infection model.

d Represents the weighted percent probability of PK-PD target attainment over the meropenem-vaborbactam MIC distribution for a given pathogen.

### Summary of PK-PD target attainment results for the U.S. FDA submission.

**(i) *Enterobacterales*.** As shown in Table 2, percent probabilities of PK-PD target attainment for the U.S. FDA submission based on the meropenem free-drug plasma %T>MIC ≥30, 35, 40, and 45% targets ranged from 99.5 to 100% and 88.4 to 100% at MIC values of 4 and 8 μg/mL, respectively, among simulated patients by eGFR group. At a MIC value of 16 μg/mL, percent probabilities of PK-PD target attainment based on meropenem free-drug plasma %T>MIC ≥30, 35, 40, and 45% targets ranged from 74.5 to 95.2%, 68.6 to 91.8%, 60.8 to 86.0%, and 52.8 to 76.1%, respectively, among simulated patients by eGFR group. Overall percent probabilities of PK-PD target attainment based on the four above-described meropenem free-drug plasma %T>MIC targets and the meropenem-vaborbactam MIC distribution for *Enterobacterales* isolates ranged from 99.5 to 99.7% among simulated patients by eGFR group.

As shown in Table 4, percent probabilities of PK-PD target attainment based on the four above-described meropenem free-drug plasma %T>MIC targets ranged from 99.8 to 100% and 96.7 to 99.7% at MIC values of 4 and 8 μg/mL, respectively, among simulated patients with cUTI or AP and various levels of renal function. At a MIC value of 16 μg/mL, percent probabilities of PK-PD target attainment based on meropenem free-drug plasma %T>MIC ≥30, 35, 40, and 45% targets were 93.9, 90.9, 86.2, and 78.9%, respectively. Overall percent probabilities of PK-PD target attainment based on the four above-described meropenem free-drug plasma %T>MIC targets and the meropenem-vaborbactam MIC distribution for *Enterobacterales* isolates ranged from 99.6 to 99.7% among simulated patients with cUTI or AP and various levels of renal function.

**(ii) Klebsiella pneumoniae** **carbapenemase-producing *Enterobacterales*.** As shown in Table 2, percent probabilities of PK-PD target attainment for the U.S. FDA submission based on the four above-described meropenem free-drug plasma %T>MIC targets ranged from 99.1 to 100% and 77.5 to 97.8% at MIC values of 4 and 8 μg/mL, respectively, among simulated patients by eGFR group. At a MIC value of 16 μg/mL, percent probabilities of PK-PD target attainment based on meropenem free-drug plasma %T>MIC ≥30, 35, 40, and 45% targets ranged from 18.3 to 49.2%, 14.9 to 45.3%, 11.4 to 42.7%, and 8.80 to 39.0%, respectively, among simulated patients by eGFR group. Overall percent probabilities of PK-PD target attainment based on the four above-described meropenem free-drug plasma %T>MIC targets and the meropenem-vaborbactam MIC distribution for KPC-producing *Enterobacterales* isolates ranged from 99.4 to 99.6% among simulated patients by eGFR group.

As shown in Table 4, percent probabilities of PK-PD target attainment based on the four above-described meropenem free-drug plasma %T>MIC targets ranged from 99.7 to 100% and 89.6 to 94.9% at MIC values of 4 and 8 μg/mL, respectively, among simulated patients with cUTI or AP and various levels of renal function. At a MIC value of 16 μg/mL, percent probabilities of PK-PD target attainment ranged from 29.7 to 46.3%. Overall percent probabilities of PK-PD target attainment based on the four above-described meropenem free-drug plasma %T>MIC targets and the meropenem-vaborbactam MIC distribution for KPC-producing *Enterobacterales* isolates ranged from 99.5 to 99.6% among simulated patients with cUTI or AP and various levels of renal function.

**(iii) Pseudomonas aeruginosa.** As shown in Table 2, percent probabilities of PK-PD target attainment for U.S. FDA submission based on the four above-described meropenem free-drug plasma %T>MIC targets ranged from 99.5 to 100% and 90.3 to 100% at MIC values of 4 and 8 μg/mL, respectively, among simulated patients by eGFR group. At a MIC value of 16 μg/mL, percent probabilities of PK-PD target attainment based on meropenem free-drug plasma %T>MIC ≥30, 35, 40, and 45% targets ranged from 76.6 to 96.0%, 71.5 to 92.7%, 63.4 to 86.7%, and 55.8 to 77.9%, respectively, among simulated patients by eGFR group. Overall percent probabilities of PK-PD target attainment based on the four above-described meropenem free-drug plasma %T>MIC targets and the meropenem-vaborbactam MIC distribution for P. aeruginosa isolates ranged from 89.9 to 95.0% among simulated patients by eGFR group.

As shown in Table 4, percent probabilities of PK-PD target attainment based on the four above-described meropenem free-drug plasma %T>MIC targets ranged from 99.8 to 100% and 97.0 to 99.8% at MIC values of 4 and 8 μg/mL, respectively, among simulated patients with cUTI or AP and various levels of renal function. At a MIC value of 16 μg/mL, percent probabilities of PK-PD target attainment based on meropenem free-drug plasma %T>MIC ≥30, 35, 40, and 45% targets were 95.1, 92.5, 88.4, and 81.2%, respectively. Overall percent probabilities of PK-PD target attainment based on the four above-described meropenem free-drug plasma %T>MIC targets and the meropenem-vaborbactam MIC distribution for P. aeruginosa isolates ranged from 92.7 to 94.9% among simulated patients with cUTI or AP and various levels of renal function.

### Summary of PK-PD target attainment results for the EMA submission.

**(i) *Enterobacterales*.** As shown in Table 3, percent probabilities of PK-PD target attainment for the EMA submission based on the meropenem free-drug plasma %T>MIC ≥30, 35, 40, and 45% targets ranged from 99.6 to 100% and 93.8 to 99.9% at MIC values of 4 and 8 μg/mL, respectively, among simulated patients by absolute eGFR group. At a MIC value of 16 μg/mL, percent probabilities of PK-PD target attainment based on meropenem free-drug plasma %T>MIC ≥30, 35, 40, and 45% targets ranged from 73.2 to 95.5%, 70.4 to 94.4%, 67.9 to 92.5%, and 64.4 to 90.0%, respectively, among simulated patients by absolute eGFR group. Overall percent probabilities of PK-PD target attainment based on the four above-described meropenem free-drug plasma %T>MIC targets and the meropenem-vaborbactam MIC distribution for *Enterobacterales* isolates ranged from 99.5 to 99.7% among simulated patients by absolute eGFR group.

As shown in Table 4, percent probabilities of PK-PD target attainment based on the four above-described meropenem free-drug plasma %T>MIC targets ranged from 99.8 to 100% and 97.4 to 99.9% at MIC values of 4 and 8 μg/mL, respectively, among simulated patients with cUTI or AP and various levels of renal function. At a MIC value of 16 μg/mL, percent probabilities of PK-PD target attainment based on meropenem free-drug plasma %T>MIC ≥30, 35, 40, and 45% targets were 95.7, 93.1, 88.7, and 81.7%, respectively. Overall percent probabilities of PK-PD target attainment based on the four above-described meropenem free-drug plasma %T>MIC targets and the meropenem-vaborbactam MIC distribution for *Enterobacterales* isolates ranged from 99.6 to 99.7% among simulated patients with cUTI or AP and various levels of renal function.

**(ii) Klebsiella pneumoniae** **carbapenemase-producing *Enterobacterales*.** As shown in Table 3, percent probabilities of PK-PD target attainment for the EMA submission based on the four above-described meropenem free-drug plasma %T>MIC targets ranged from 99.0 to 100% and 81.2 to 99.7%, respectively, among simulated patients by absolute eGFR group at MIC values of 4 and 8 μg/mL, respectively, among simulated patients by absolute eGFR group. At a MIC value of 16 μg/mL, percent probabilities of PK-PD target attainment based on meropenem free-drug plasma %T>MIC ≥30, 35, 40, and 45% targets ranged from 28.5 to 81.5%, 27.4 to 79.7%, 25.4 to 78.0%, and 21.8 to 75.6%, respectively, among simulated patients by absolute eGFR group. Overall percent probabilities of PK-PD target attainment based on the four above-described meropenem free-drug plasma %T>MIC targets and the meropenem-vaborbactam MIC distribution for KPC-producing *Enterobacterales* isolates ranged from 99.5 to 99.7% among simulated patients by absolute eGFR group.

As shown in Table 4, percent probabilities of PK-PD target attainment based on the four above-described meropenem free-drug plasma %T>MIC targets ranged from 99.7 to 100% and 91.0 to 95.9% at MIC values of 4 and 8 μg/mL, respectively, among simulated patients with cUTI or AP and various levels of renal function. At a MIC value of 16 μg/mL, percent probabilities of PK-PD target attainment ranged from 36.0 to 52.9%. Overall percent probabilities of PK-PD target attainment based on the four above-described meropenem free-drug plasma %T>MIC targets and the meropenem-vaborbactam MIC distribution for KPC-producing *Enterobacterales* isolates was 99.6% across %T>MIC targets among simulated patients with cUTI or AP and various levels of renal function.

**(iii) Pseudomonas aeruginosa.** As shown in Table 3, percent probabilities of PK-PD target attainment for the EMA submission based on the four above-described meropenem free-drug plasma %T>MIC targets ranged from 99.7 to 100% and 95.4 to 99.9% at MIC values of 4 and 8 μg/mL, respectively, among simulated patients by absolute eGFR group. At a MIC value of 16 μg/mL, percent probabilities of PK-PD target attainment based on meropenem free-drug plasma %T>MIC ≥30, 35, 40, and 45% targets ranged from 75.9 to 96.4%, 73.4 to 94.7%, 70.7 to 92.9%, and 67.2 to 90.7%, respectively, among simulated patients by absolute eGFR group. Overall percent probabilities of PK-PD target attainment based on the four above-described meropenem free-drug plasma %T>MIC targets and the meropenem-vaborbactam MIC distribution for P. aeruginosa isolates ranged from 91.2 to 95.2% among simulated patients by absolute eGFR group.

As shown in Table 4, percent probabilities of PK-PD target attainment based on the four above-described meropenem free-drug plasma %T>MIC targets ranged from 99.8 to 100% and 97.6 to 99.9% at MIC values of 4 and 8 μg/mL, respectively, among simulated patients with cUTI or AP and various levels of renal function. At a MIC value of 16 μg/mL, percent probabilities of PK-PD target attainment based on meropenem free-drug plasma %T>MIC ≥30, 35, 40, and 45% targets were 96.6, 94.4, 90.6, and 83.9%, respectively. Overall percent probabilities of PK-PD target attainment based on the four above-described meropenem free-drug plasma %T>MIC targets and the meropenem-vaborbactam MIC distribution for P. aeruginosa isolates ranged from 93.0 to 95.2% among simulated patients with cUTI or AP and various levels of renal function.

## DISCUSSION

The objective of the PK-PD target attainment analyses described herein was to evaluate 2 g meropenem–2 g vaborbactam q8h administered as a 3-h i.v. infusion and dosing regimens for patients with renal impairment. A second objective of these analyses was to provide support for decisions about interpretive criteria for the *in vitro* susceptibility testing of meropenem-vaborbactam (using a fixed vaborbactam concentration of 8 μg/mL) against *Enterobacterales* and P. aeruginosa. These analyses were undertaken using population PK models for meropenem and vaborbactam (10), nonclinical PK-PD targets for efficacy (2, 11–13), *in vitro* surveillance data (1, 14–16), and simulation.

At a meropenem-vaborbactam MIC value of 4 μg/mL, 80.4 to 99.3% of the collections of *Enterobacterales*, KPC-producing *Enterobacterales*, and P. aeruginosa isolates (1, 14–16) were inhibited. The percent probabilities of achieving the meropenem free-drug plasma %T>MIC target of ≥40% and vaborbactam free-drug plasma AUC:MIC ratio target of 38, each of which were associated with a 1-log_10_ CFU reduction from baseline, at this MIC value ranged from 99.4 to 100% and 99.2 to 100% among simulated patients by renal function group for the U.S. FDA and EMA submissions, respectively. At a MIC value of 8 μg/mL, 86.4 to 99.5% of isolates (1, 14–16) were inhibited, with corresponding percent probabilities of PK-PD target attainment that ranged from 81.3 to 99.3% and 84.6 to 99.9% for the respective submissions. Overall percent probabilities of PK-PD target attainment based on the above-described PK-PD targets and MIC distributions ranged from 90.8 to 99.6% and 91.6 to 99.7% among simulated patients by renal function group for the U.S. FDA and EMA submissions, respectively.

In addition to assessing the meropenem-vaborbactam dosing regimens among simulated patients by renal function group, these dosing regimens were assessed in simulated patients with cUTI or AP and various levels of renal function that were generated using the observed clinical data from the TANGO I and II studies (8, 9). When the same PK-PD targets were assessed among these simulated patients, percent probabilities of PK-PD target attainment ranged from 99.9 to 100% at a MIC value of 4 μg/mL across assessments for the pathogen groups for both submissions. At a meropenem-vaborbactam MIC value of 8 μg/mL, percent probabilities of PK-PD target attainment ranged from 92.0 to 98.8% and 93.2 to 99.1% for the U.S. FDA and EMA submissions, respectively. Overall percent probabilities of PK-PD target attainment based on the MIC distributions assessed ranged from 93.6 to 99.6% and 94.0 to 99.7% for the U.S. FDA and EMA submissions, respectively.

The results of these analyses provided support for 2 g meropenem-2 g vaborbactam q8h administered as a 3-h i.v. infusion to patients with normal renal function and dosing regimens for patients with renal impairment As shown in Table 1, recommendations for the use of these dosing regimens by region vary only by the renal function measure used (U.S. FDA: eGFR in mL/min/1.73 m^2^ [20]; EMA: absolute eGFR in mL/min) and the differences in ranges of each measure that guided dose adjustment. While the package inserts by region reflect these recommendations, those for the EMA were based on the method described by Cockcroft and Gault (creatine clearance [CLcr], mL/min) (21) rather than absolute eGFR. The results of these analyses also provide support for both meropenem-vaborbactam susceptibility breakpoints of 4 and 8 μg/mL based on these dosing regimens.

The results of the analyses described herein were used to support proposals for interpretive criteria for the *in vitro* susceptibility testing of meropenem-vaborbactam against *Enterobacterales* and P. aeruginosa in review packages for the U.S. FDA and later by the Clinical and Laboratory Standards Institute (CLSI), the United States Committee on Antimicrobial Susceptibility Testing (USCAST), and the European Committee on Antimicrobial Susceptibility Testing (EUCAST). At the time of approval, the FDA interpretive criteria for the *in vitro* susceptibility testing of meropenem-vaborbactam against *Enterobacterales* were ≤4/8, 8/8, and ≥16/8 μg/mL for susceptible, intermediate, and resistant, respectively (22). These interpretive criteria were subsequently endorsed by CLSI (17). In contrast, both USCAST and EUCAST granted interpretive criteria for meropenem-vaborbactam against *Enterobacterales* of ≤8/8 and ≥16/8 μg/mL (the latter denoted as >8/8 μg/mL for EUCAST) for susceptible and resistant, respectively. For P. aeruginosa, only USCAST and EUCAST established meropenem-vaborbactam interpretive criteria, defining susceptible as ≤8/8 μg/mL and resistant as ≥16/8 μg/mL (18, 19). It is important to note that the data described herein also provide support for the interpretive criteria for the *in vitro* susceptibility testing of meropenem against P. aeruginosa since vaborbactam has not been shown to improve the activity of meropenem for this pathogen (15). Currently, the CLSI meropenem interpretive criteria define susceptible, intermediate, and resistant as ≤2, 4, and ≥8 μg/mL, respectively, based on a meropenem dosing regimen of 1 g i.v. q8h (17). USCAST and EUCAST meropenem interpretive criteria define susceptible as ≤2 and resistant as ≥8 μg/mL for USCAST and ≥16 μg/mL for EUCAST (18, 19), with the former organization describing the standard meropenem 1 g i.v. q8h dosing regimen upon which these criteria are based. Thus, the results of the PK-PD target attainment analyses described herein for P. aeruginosa provide support for changes to the above-described meropenem interpretive criteria based on the administration of 2 g meropenem q8h administered as a 3-h i.v. infusion.

There can be challenges in gathering sufficient clinical data for multidrug-resistant (MDR) pathogens with MIC values near those for PK-PD-derived breakpoints, particularly when the dosing regimens for new drugs provide exposures that will cover a large proportion of clinical isolates. Noninferiority studies, such as TANGO I (8), which compare the investigational agent to a standard agent, require that the infecting isolate for enrolled patients be susceptible to both agents. In this circumstance, even if the investigational agent is active against MDR pathogens, the number of patients enrolled with elevated MIC values will be limited given the lack of such activity for the standard agent. Clinical studies that compare a new antibiotic that is active against MDR pathogens to “best available therapy” are difficult to enroll and thus, may be small studies that may not include pathogens with a MIC value near potential PK-PD-derived breakpoints. For the TANGO II study, a phase 3, randomized open-label study of meropenem-vaborbactam versus the best available therapy for the treatment of patients with infections due to confirmed or suspected carbapenem-resistant *Enterobacterales*, meropenem-vaborbactam was associated with higher rates of clinical cure at end of treatment and test of cure in the microbiological carbapenem-resistant *Enterobacterales* modified intent-to-treat population. Ultimately, for ethical reasons, the trial was stopped because meropenem-vaborbactam was found to be superior to the “best available therapy” group (9, 23).

For both of the above-described study designs, there were few isolates with elevated MIC values to the investigational therapy to enable evaluations of clinical and microbiological outcomes for isolates with MIC values near the potential susceptibility breakpoints of 4 or 8 μg/mL. Given such limited data, regulatory bodies and some standards development organizations have been reluctant to set susceptibility breakpoints above the highest MIC values observed in clinical trials, despite supportive PK-PD data. This creates a perverse incentive such that future drug developers may be reluctant to develop antibiotic dosing regimens with sufficient exposures to treat pathogens at the upper margins of the MIC distribution. As a result, patients will be denied likely effective antimicrobial dosing regimens. Given that isolates with elevated meropenem-vaborbactam MIC values have emerged in parts of the world (24, 25), the FDA and CLSI interpretative criteria (17, 26) should be reevaluated.

In conclusion, the results of PK-PD target attainment analyses were used to support 2 g meropenem-2 g vaborbactam q8h administered as a 3-h i.v. infusion, as well as dose adjustments for renal function for patients with cUTI or AP that are described in the U.S. FDA and EMA labels (3, 4). Lastly, and equally important, the results of these analyses were used to justify a higher susceptibility breakpoint than was supported by the pivotal clinical trial data alone. A higher susceptibility breakpoint for meropenem-vaborbactam is critical to ensuring use of this combination therapy in patients infected with isolates with higher MIC values, the occurrence of which has been reported in global regions (24, 25).

## MATERIALS AND METHODS

The process to generate simulated patients and exposures for these simulated patients (including brief descriptions of the population PK models for meropenem and vaborbactam [10] used), the meropenem-vaborbactam dosing regimens evaluated, and the approach for assessing percent probabilities of PK-PD target attainment, including the nonclinical PK-PD targets for efficacy (2, 11–13) and *in vitro* surveillance data (1, 14–16) evaluated, are described below.

### Generation of simulated patients.

Using R version 3.3.1 (27), two sets of simulations were performed for both the U.S. FDA and EMA submissions. For the first simulation for each submission, populations of 5,000 simulated patients with various degrees of renal function were generated. For the U.S. FDA submission, eGFR (mL/min/1.73 m^2^) values, calculated using the modification of diet in renal function equation (20), were generated using a uniform probability distribution for the following renal function groups, each of which contained 1,000 simulated patients: ≥0 to <15, ≥15 to <30, ≥30 to <50, ≥50 to <150, and ≥150 to ≤200 mL/min/1.73 m^2^. For the EMA submission, absolute eGFR (mL/min) values (which are the product of eGFR for an individual simulated patient and that individual’s body surface area divided by 1.73 m^2^) were also generated using a uniform probability distribution and the same sample size per group for the following renal function groups: ≥0 to <10, ≥10 to <20, ≥20 to <40, ≥40 to <150, and ≥150 to ≤200 mL/min. Demographics for each group were generated by randomly sampling with replacement 1,000 patients from the phase 3 PK analysis population consisting of 295 patients with cUTI or AP (10). As described below, these eGFR or absolute eGFR groupings were based on prior analyses to determine the most appropriate eGFR or absolute eGFR thresholds for meropenem-vaborbactam dose adjustment.

For the second simulation for each submission, an analysis population based on data from two phase 3 studies, TANGO I and II, used to develop the population PK models (8, 9) and consisting of 295 patients with cUTI or AP (10), was used to generate a simulated clinical population. The simulated patient population was generated by replicating each patient record (i.e., the demographics for each patient) 11 times to achieve a total sample size of 3,245. The choice of the number of records for the above-described simulated clinical trial population was based on the goal of achieving a total sample size of at least 3,000 simulated patients. For both sets of simulations, those assessed by renal function group or those with various levels of renal function and resembling the clinical trial populations, patient PK parameters for meropenem and vaborbactam were calculated for each simulated patient using demographic values and population PK models for each agent (10).

### Generation of meropenem and vaborbactam exposures for simulated patients.

For both agents, a two-compartment model with first-order elimination best described the PK data with a relationship between renal clearance and eGFR incorporated. For meropenem, the following significant covariate relationships were identified: clearance (CL) with age, CL with end-stage renal disease, and volume of the central compartment (Vc) and volume of the peripheral compartment (Vp) with weight. For vaborbactam, the following significant covariate relationships were identified: CL with height, Vc with body surface area, and CL, Vc, and Vp with health status. The population PK models were subsequently refined using a pooled data set that included data used to develop the original model and additional data from infected patients from the TANGO II study after study completion. Updates to the meropenem population PK model included applying allometric scaling, incorporating interindividual variability on distributional clearance, and using a full covariance matrix. Updates to the vaborbactam population PK model only included using a full covariance matrix (10).

Using the above-described individual PK parameters and population PK models (10), total-drug concentration-time profiles for meropenem and vaborbactam after administration of the meropenem-vaborbactam dosing regimens evaluated were generated for each simulated patient. These profiles were generated from 0 to 24 h on day 1 and at steady state. Using a plasma protein binding value of 2% for meropenem (3, 4), the meropenem free-drug plasma %T>MIC was then determined by counting the total number of free-drug plasma concentrations that were above a given MIC value, multiplying this number by the time interval between simulated concentrations (0.1 h), and then dividing this product by 24 h. For vaborbactam, total-drug plasma AUC values were calculated by numerical integration of the total-drug plasma concentration-time profiles. Using a plasma protein binding estimate of 33% for vaborbactam (3, 4), vaborbactam total-drug plasma AUC values were adjusted to free-drug plasma AUC values. Vaborbactam free-drug plasma AUC:MIC ratios were determined by dividing free-drug plasma AUC values by fixed meropenem-vaborbactam MIC values.

Meropenem-vaborbactam dosing regimens for simulated patients with renal impairment were based on those evaluated in the TANGO I and II studies (8, 9). Dosing regimens administered to patients in these studies were evaluated and refinements were considered necessary. The objective was to identify meropenem-vaborbactam dosing regimens by the above-described eGFR or absolute eGFR groups that best matched day 1 and steady-state meropenem and vaborbactam free-drug plasma AUC_0–24_ values for simulated patients with mild renal impairment to normal or augmented renal function (eGFR of ≥50 to <150 and ≥150 to ≤200 mL/min/1.73 m^2^ or absolute eGFR of ≥40 to <150 and ≥150 to ≤200 mL/min).

### PK-PD targets for efficacy.

Meropenem free-drug plasma %T>MIC targets of 30, 35, 40, and 45%, the minimum and maximum values of which encompassed those associated with net bacterial stasis and 1- and 2-log_10_ CFU reductions from baseline for Gram-negative bacilli, were evaluated. These %T>MIC targets were based on data for carbapenem agents studied in a neutropenic murine-thigh infection model (11–13). For vaborbactam, a free-drug plasma AUC:MIC ratio of 38, which is associated with a 1-log_10_ CFU reduction from baseline, was evaluated. This PK-PD index was calculated using the meropenem-vaborbactam MIC value (i.e., using the meropenem MIC value tested with vaborbactam at 8 μg/mL). The vaborbactam free-drug plasma AUC:MIC ratio target was determined using a neutropenic murine-thigh infection model for KPC-producing *Enterobacterales* and a fixed exposure of meropenem (2).

### Assessment of PK-PD target attainment.

Using the above-described PK-PD targets for meropenem and vaborbactam, the algorithm shown in Fig. S4 was used to calculate percent probabilities of PK-PD target attainment by MIC and overall (i.e., weighted over a given MIC distribution) for the meropenem-vaborbactam dosing regimens described in Table 1. This algorithm involved the assessment of PK-PD target attainment by the meropenem and corresponding meropenem-vaborbactam MIC values, based on *in vitro* surveillance data for *Enterobacterales* and KPC-producing *Enterobacterales* (1, 14–16).

This algorithm considered whether the meropenem free-drug plasma %T>MIC target could be achieved based on meropenem free-drug plasma exposures and the meropenem MIC value. If this was the case, the PK-PD target attainment based on the vaborbactam free-drug plasma exposure and above-described AUC:MIC ratio target was not considered. However, if the meropenem free-drug plasma %T>MIC target could not be achieved based on meropenem free-drug plasma exposure and the meropenem MIC, the meropenem-vaborbactam MIC and PK-PD target attainment of meropenem and vaborbactam was considered.

For the evaluation of percent probabilities of PK-PD target attainment by MIC for P. aeruginosa, simulated patients were considered in the context of achieving the above-described meropenem free-drug plasma %T>MIC targets relative to the meropenem-vaborbactam MIC value without consideration of vaborbactam exposure. Meropenem-vaborbactam MIC was chosen as the reference measure for *in vitro* activity since meropenem and meropenem-vaborbactam MIC distributions for P. aeruginosa are similar based on *in vitro* surveillance data (15).

In addition to their use to assess PK-PD target attainment, the MIC distributions for meropenem and meropenem-vaborbactam against *Enterobacterales*, KPC-producing *Enterobacterales*, and P. aeruginosa isolates were used to calculate overall percent probabilities of PK-PD target attainment and interpret the PK-PD target attainment results. These MIC distributions are summarized in Table S3. Clinical *Enterobacterales*, KPC-producing *Enterobacterales*, and P. aeruginosa isolates were collected worldwide from patients in 2014 to 2015 by JMI Laboratories and from International Health Management Associates (IHMA) (1, 14–16).

For each set of meropenem and vaborbactam free-drug plasma PK-PD targets for a given bacterial reduction target endpoint and MIC distribution, the overall percent probability of PK-PD target attainment was determined by multiplying the percent probability of PK-PD target attainment at each MIC value with the probability of occurrence of that MIC value. The sum of these products was then determined.

For decisions about meropenem-vaborbactam dosing regimens for patients with cUTI or AP, emphasis was placed on the results of PK-PD target attainment analyses based on free-drug plasma PK-PD targets associated with net bacterial stasis and a 1-log_10_ CFU reduction from baseline.
