# Supplementary material for: Pharmacokinetic-Pharmacodynamic Target Attainment Analyses as Support for Meropenem-Vaborbactam Dosing Regimens and Susceptibility Breakpoints
Source: Antimicrob Agents Chemother. 2022 Nov 14;66(12):e02130-21. doi: 10.1128/aac.02130-21 (PMC9764998; doi:10.1128/aac.02130-21)
Supplement: Supplemental file 1 — Tables S1 to S3 and Fig. S1 to S4. Download aac.02130-21-s0001.pdf, PDF file, 2.3 MB [file aac.02130-21-s0001.pdf]

Table S1. Summary statistics for meropenem and vaborbactam free-drug plasma AUC_{0-24} on day 1 and at steady state among simulated patients with cUTI or AP by renal function group for the U.S. FDA and EMA submissions

Free-drug plasma AUC_{0-24} measure (mg•h/L)	US FDA submission						EMA submission					
	eGFR (mL/min/ 1.73 m^2)	Mean	%CV	Min	Median	Max	Absolute eGFR (mL/min)	Mean	%CV	Min	Median	Max
Day 1 free-drug plasma meropenem AUC_{0-24}	≥0 to <15	479.06	52.80	82.62	426.30	2600.51	≥0 to <10	496.9	51.25	66.38	444.0	2009
	≥15 to <30	638.85	53.22	93.61	569.41	2631.94	≥10 to <20	787.3	49.37	116.6	713.3	3105
	≥30 to <50	432.36	51.79	89.14	384.08	1612.30	≥20 to <40	690.4	58.10	115.5	606.6	3548
	≥50 to <150	675.07	56.92	113.26	601.98	4384.92	≥40 to <150	713.3	54.08	113.7	628.6	2943
	≥150 to ≤200	624.72	52.75	95.83	554.89	2089.70	≥150 to ≤200	631.8	55.60	90.37	551.5	2952
Steady-state free-drug plasma meropenem AUC_{0-24}	≥0 to <15	681.81	62.28	93.85	595.44	4135.80	≥0 to <10	735.3	57.80	92.68	626.2	2897
	≥15 to <30	748.55	58.51	94.92	653.14	3825.05	≥10 to <20	999.0	53.00	126.8	878.9	3736
	≥30 to <50	453.20	53.26	89.49	399.71	1684.04	≥20 to <40	808.2	66.35	115.7	696.3	4390
	≥50 to <150	693.80	58.02	113.40	614.17	4589.98	≥40 to <150	735.7	55.27	114.9	642.9	3140
	≥150 to ≤200	639.63	53.74	95.89	566.70	2292.44	≥150 to ≤200	647.2	56.67	91.53	560.9	3230
Day 1 free-drug plasma vaborbactam AUC_{0-24}	≥0 to <15	533.03	37.79	142.79	499.88	1539.74	≥0 to <10	562.7	36.65	146.8	532.4	1549
	≥15 to <30	669.97	37.67	146.42	627.10	2108.92	≥10 to <20	891.1	33.10	294.0	857.0	2420
	≥30 to <50	531.31	41.09	120.50	492.37	1417.95	≥20 to <40	737.2	38.29	172.1	695.6	2093
	≥50 to <150	605.15	44.69	147.58	559.30	1848.97	≥40 to <150	658.2	46.16	148.6	603.4	1860
	≥150 to ≤200	510.40	42.39	91.46	475.07	1810.01	≥150 to ≤200	514.1	41.97	92.57	479.3	1798
Steady-state free-drug plasma vaborbactam AUC_{0-24}[a]	≥0 to <15	2868.31	71.53	300.76	2328.80	14508.97	≥0 to <10	3653	57.02	692.3	3297	15936
	≥15 to <30	1179.35	62.54	146.92	999.11	8029.06	≥10 to <20	2508	62.15	311.2	2146	16482
	≥30 to <50	673.72	53.52	121.92	590.38	2441.44	≥20 to <40	1195	63.66	177.9	989.1	7188
	≥50 to <150	656.29	51.32	147.97	593.58	2399.32	≥40 to <150	733.4	56.16	149.1	645.5	3519
	≥150 to ≤200	540.21	47.32	91.46	494.73	1925.72	≥150 to ≤200	544.9	47.03	92.57	499.1	1911

a. To assess the appropriateness of the steady-state free-drug plasma vaborbactam AUC_{0-24} values in simulated patients with low eGFR or absolute eGFR groups, steady-state free-drug plasma vaborbactam AUC_{0-24} values in patients with such low eGFR or absolute eGFR values from the TANGO II study included in the population PK dataset were examined. To facilitate comparisons between observed and simulated patients, individual post-hoc PK parameter estimates using baseline measures of renal function were used to generate the steady-state exposures for observed patients. Results of this comparison demonstrated similar trends for exposures in simulated and observed patients.

Table S2. Summary statistics for meropenem and vaborbactam free-drug plasma AUC_{0-24} on day 1 and at steady state among simulated patients with cUTI/AP for the U.S. FDA and EMA submissions[a]

**Drug and period or baseline renal function measure**		**Free-drug plasma AUC_{0-24} (mg•h/L) for simulated patients with cUTI/AP**									
		**US FDA submission**					**EMA submission**				
		**Mean**	**%CV**	**Min**	**Median**	**Max**	**Mean**	**%CV**	**Min**	**Median**	**Max**
Meropenem	Day 1	674	54.4	94.7	595	5039	706	52.9	94.7	628	5039
	Steady-state	701	55.3	94.7	616	5340	739	54.9	94.7	652	5340
Vaborbactam	Day 1	606	45.4	108	554	2368	642	47.3	108	581	2794
	Steady-state	707	62.7	108	600	5706	771	75.8	108	620	8300
Baseline eGFR (mL/min/1.73 m^2)		84.5	36.6	4.3	83.8	207					
Baseline absolute eGFR (mL/min)							87.9	36.0	5.0	89.3	266

a. All simulated patients (n=3,245) received meropenem-vaborbactam dosing regimens by eGFR or absolute eGFR as described in **Table 1.** Simulated patients with cUTI or AP and eGFR or absolute eGFR > 200 mL/min were assigned the same meropenem-vaborbactam dosing regimen as those with eGFR or absolute eGFR ≥ 150 to ≤ 200 mL/min.

Table S3. Meropenem and meropenem-vaborbactam MIC distributions for Enterobacterales, KPC-producing Enterobacterales, and *P. aeruginosa* isolates based on *in vitro* surveillance data collected from regions worldwide

Drug	Number of isolates at MIC (µg/mL; cumulative %)[a]														
	<0.03	0.03	0.06	0.12	0.25	0.5	1	2	4	8	16	32	>32	MIC_{50}	MIC_{90}
**All Enterobacterales (n=11,559)[b]**															
Meropenem	5595 (48.4)	3799 (81.3)	1321 (92.7)	338 (95.6)	73 (96.3)	44 (96.6)	36 (96.9)	44 (97.3)	48 (97.7)	44 (98.1)	62 (98.7)	48 (99.1)	107 (100)	0.03	0.06
Meropenem-vaborbactam	4551 (39.4)	5193 (84.3)	1208 (94.7)	271 (97.1)	89 (97.9)	69 (98.5)	50 (98.9)	28 (99.1)	14 (99.3)	9 (99.3)	22 (99.5)	32 (99.8)	23 (100)	0.03	0.06
**All KPC-producing Enterobacterales (n=1,331)[b]**															
Meropenem	–	–	–	–	–	–	5 (0.40)	58 (4.70)	116 (13.4)	159 (25.4)	200 (40.4)	179 (53.9)	614 (100)	32	>32
Meropenem-vaborbactam	515 (38.7)	68 (43.8)	78 (49.7)	89 (56.3)	195 (71.0)	186 (85.0)	110 (93.2)	55 (97.4)	22 (99.0)	7 (99.5)	2 (99.7)	1 (99.8)	3 (100)	0.12	1
**All *P. aeruginosa* (n=2,806)[b]**															
Meropenem	15.0 (0.50)	47.0 (2.20)	194 (9.10)	321 (20.6)	540 (39.8)	477 (56.8)	293 (67.2)	193 (74.1)	170 (80.2)	189 (86.9)	173 (93.1)	64 (95.4)	130 (100)	0.50	16
Meropenem-vaborbactam	30 (1.10)	65 (3.40)	193 (10.3)	310 (21.3)	525 (40.0)	462 (56.5)	298 (67.1)	186 (73.7)	187 (80.4)	167 (86.4)	187 (93.0)	71 (95.5)	125 (100)	0.50	16

a. Shaded cells represent the MIC values up to and/or including the MIC_{90} value.
b. Enterobacterales, KPC-producing Enterobacterales, and *P. aeruginosa* isolates were collected as part of the 2014-2015 SENTRY Antimicrobial Surveillance Program [1, 2, 3, 4].

Figure S1. Percent probabilities of PK-PD target attainment by meropenem-vaborbactam MIC on day 1 based on the assessment of the meropenem free-drug plasma %T>MIC ≥ 30% target and MIC data for collections of isolates among simulated patients with cUTI or AP by eGFR and absolute eGFR groups after administration of meropenem-vaborbactam dosing regimens for the U.S. FDA and EMA submissions, overlaid on the meropenem-vaborbactam MIC distributions for Enterobacterales (top), KPC-producing Enterobacterales (middle), and *P. aeruginosa* (bottom) isolates

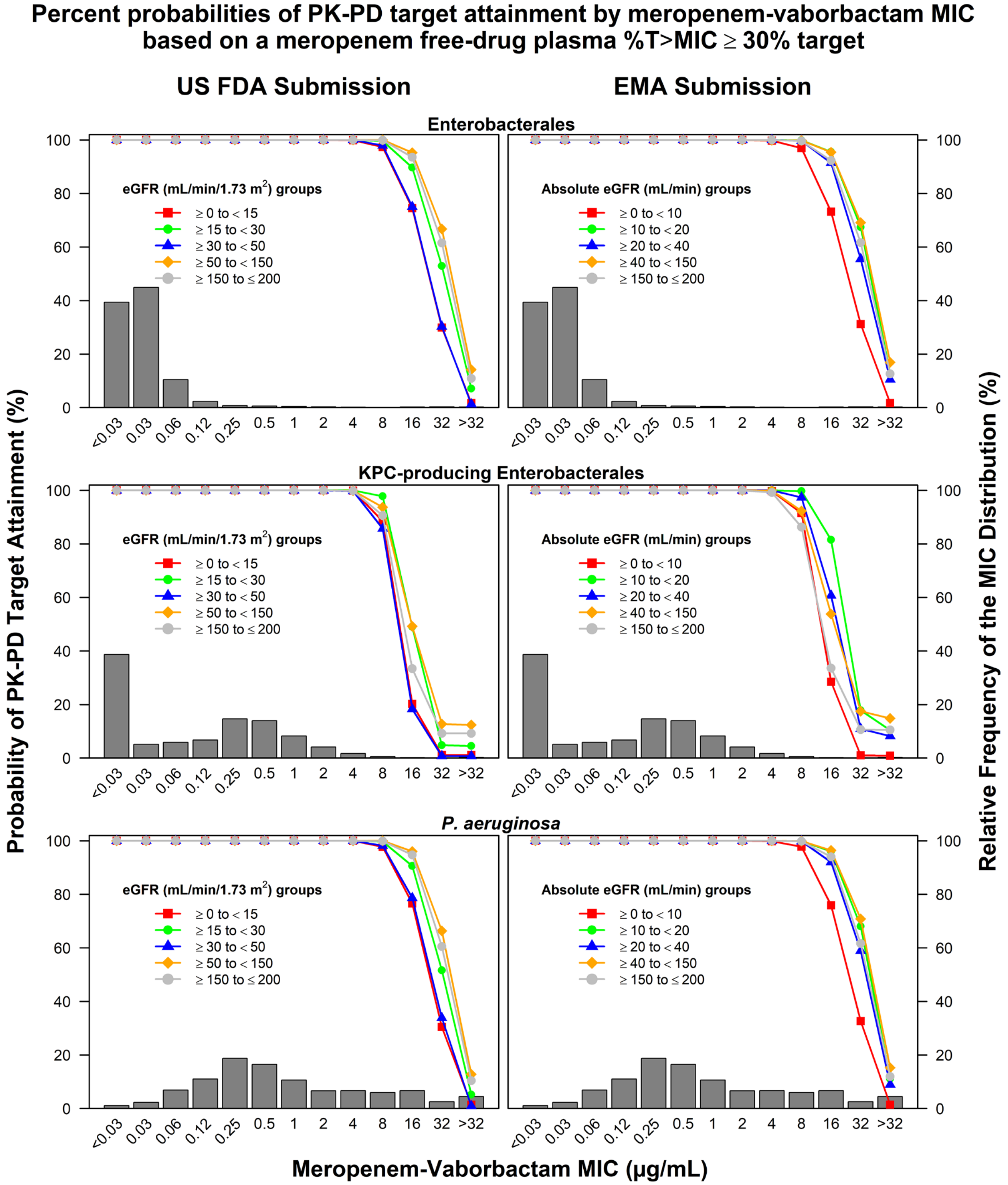

Figure S2. Percent probabilities of PK-PD target attainment by meropenem-vaborbactam MIC on day 1 based on the assessment of the meropenem free-drug plasma %T>MIC ≥ 35% target and MIC data for collections of isolates among simulated patients with cUTI or AP by eGFR and absolute eGFR groups after administration of meropenem-vaborbactam dosing regimens for the U.S. FDA and EMA submissions, overlaid on the meropenem-vaborbactam MIC distributions for Enterobacterales (top), KPC-producing Enterobacterales (middle), and *P. aeruginosa* (bottom) isolates

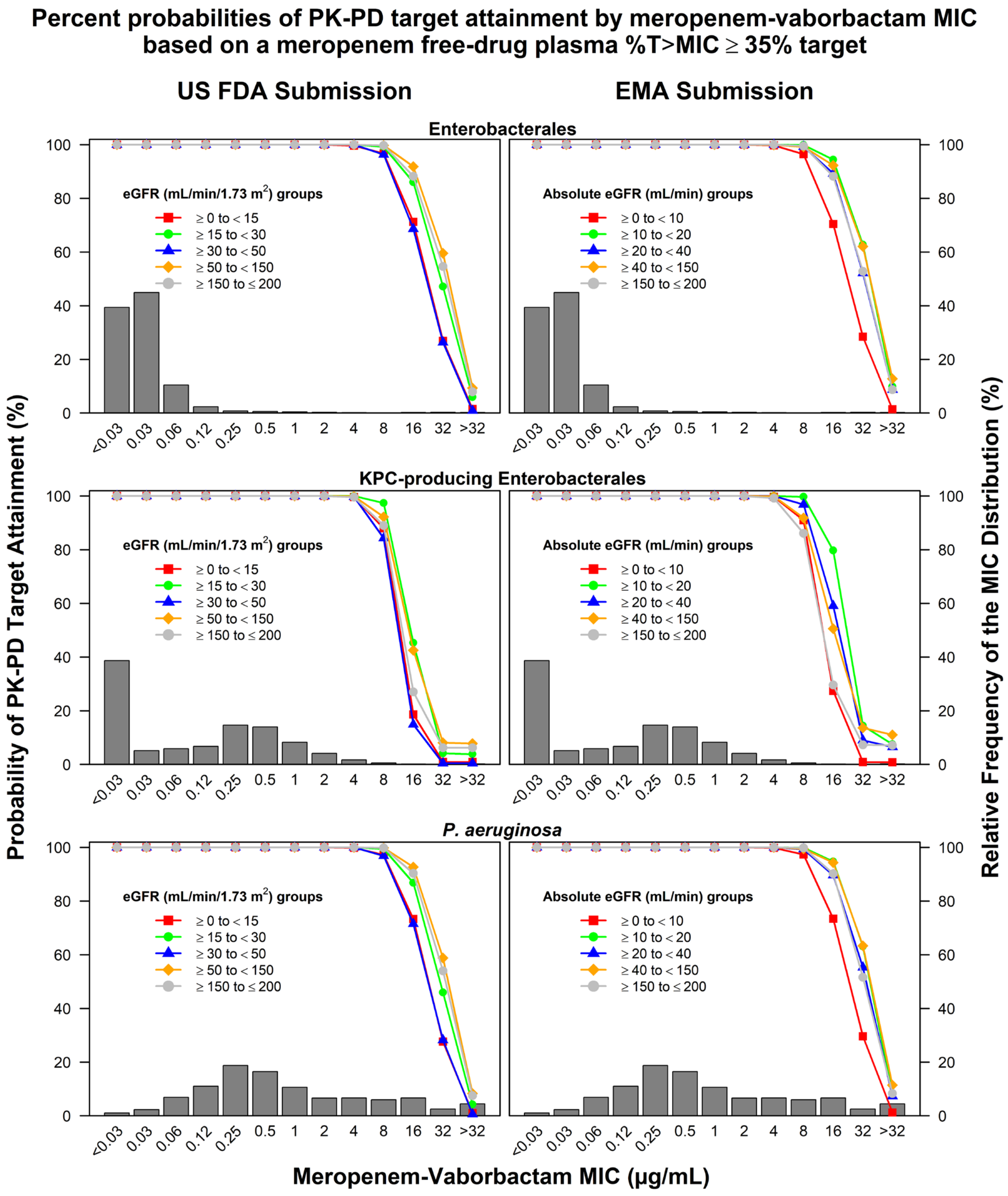

Figure S3. Percent probabilities of PK-PD target attainment by meropenem-vaborbactam MIC on day 1 based on the assessment of the meropenem free-drug plasma %T>MIC ≥ 45% target and MIC data for collections of isolates among simulated patients with cUTI or AP by eGFR and absolute eGFR groups after administration of meropenem-vaborbactam dosing regimens for the U.S. FDA and EMA submissions, overlaid on the meropenem-vaborbactam MIC distributions for Enterobacterales (top), KPC-producing Enterobacterales (middle), and *P. aeruginosa* (bottom) isolates

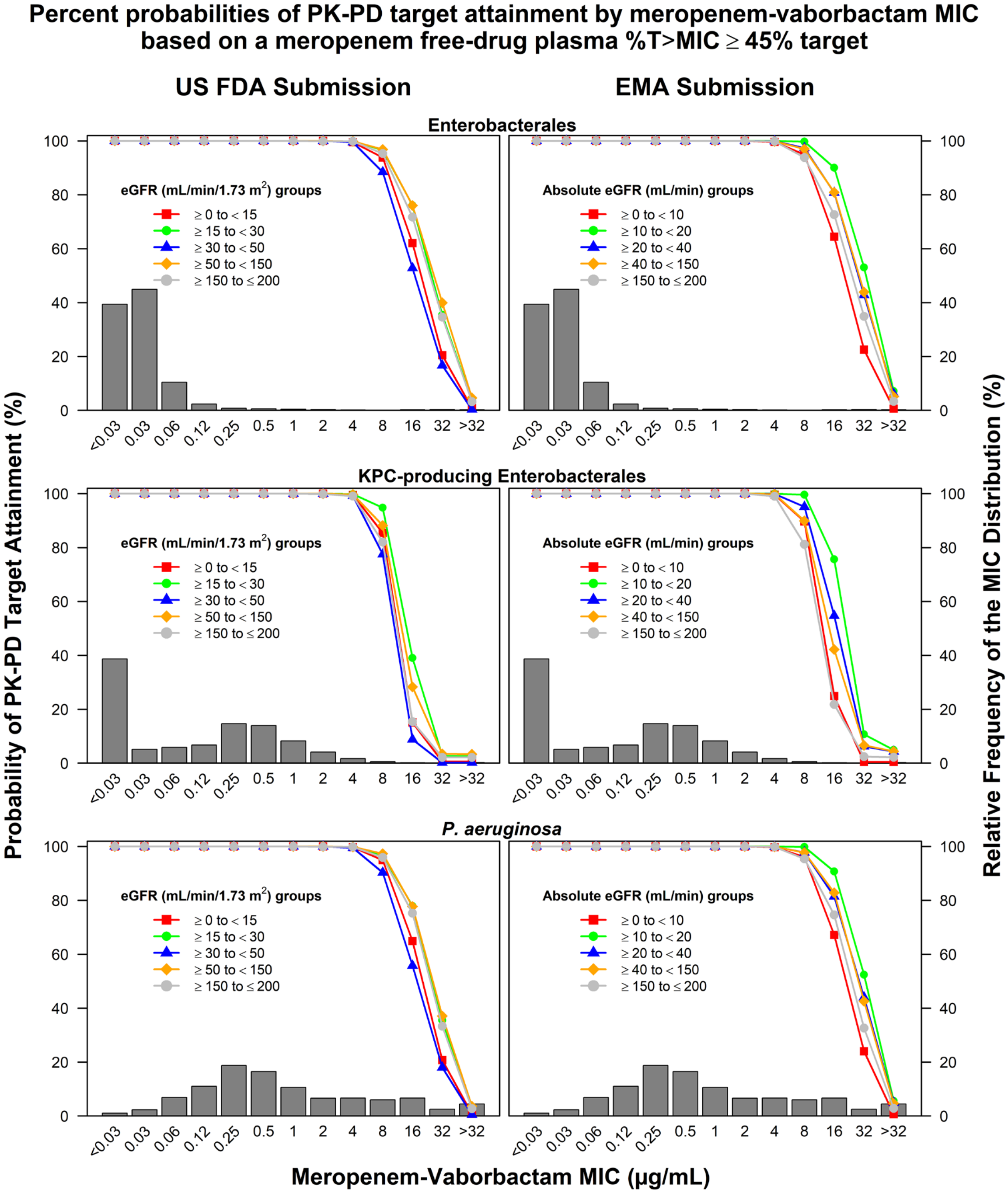

Explanation for Figure S4

The steps of the algorithm, which were applied by using sets of meropenem and vaborbactam free-drug plasma PK-PD targets associated with net bacterial stasis and 1- and 2-log_{10} CFU reductions from baseline for the assessment of meropenem-vaborbactam dosing regimens, are described below.

- If the meropenem MIC value for the isolate was less than or equal to the meropenem-vaborbactam MIC value, then the given meropenem free-drug plasma %T>MIC was calculated using the meropenem MIC value and compared to the meropenem free-drug plasma %T>MIC target for the same bacterial reduction endpoint.
    - If the calculated meropenem free-drug plasma %T>MIC was at least equal to the meropenem free-drug plasma %T>MIC target, PK-PD target attainment was classified as achieved,
    - and if not, as not achieved.
- If the meropenem MIC value for the isolate was greater than the meropenem-vaborbactam MIC value, the given meropenem free-drug plasma %T>MIC was calculated using the meropenem MIC value and compared to the meropenem free-drug plasma %T>MIC target.
    - If the calculated meropenem free-drug plasma %T>MIC was at least equal to the meropenem free-drug plasma %T>MIC target, PK-PD target attainment was classified as achieved.
    - If not, the vaborbactam free-drug plasma AUC:MIC ratio (calculated using the meropenem-vaborbactam MIC value) was calculated in order to assess whether or not the meropenem-vaborbactam, MIC value can be used.
        - If the calculated vaborbactam free-drug plasma AUC:MIC ratio was at least equal to the vaborbactam free-drug plasma AUC:MIC ratio target for a given endpoint, the meropenem free-drug plasma %T>MIC (using the meropenem-vaborbactam MIC value) was calculated and compared to the meropenem free-drug plasma %T>MIC target.
            - If the calculated meropenem free-drug plasma %T>MIC was at least equal to the meropenem free-drug plasma %T>MIC target, PK-PD target attainment was classified as achieved,
            - and if not, as not achieved.
        - If the calculated vaborbactam free-drug plasma AUC:MIC ratio was less than the vaborbactam free-drug plasma AUC:MIC ratio target, PK-PD target attainment was classified as not achieved (given that the meropenem free-drug plasma %T>MIC target had not been achieved using the meropenem MIC value).

Figure S4. Algorithm to calculate percent probabilities of PK-PD target attainment for meropenem-vaborbactam dosing regimens evaluated

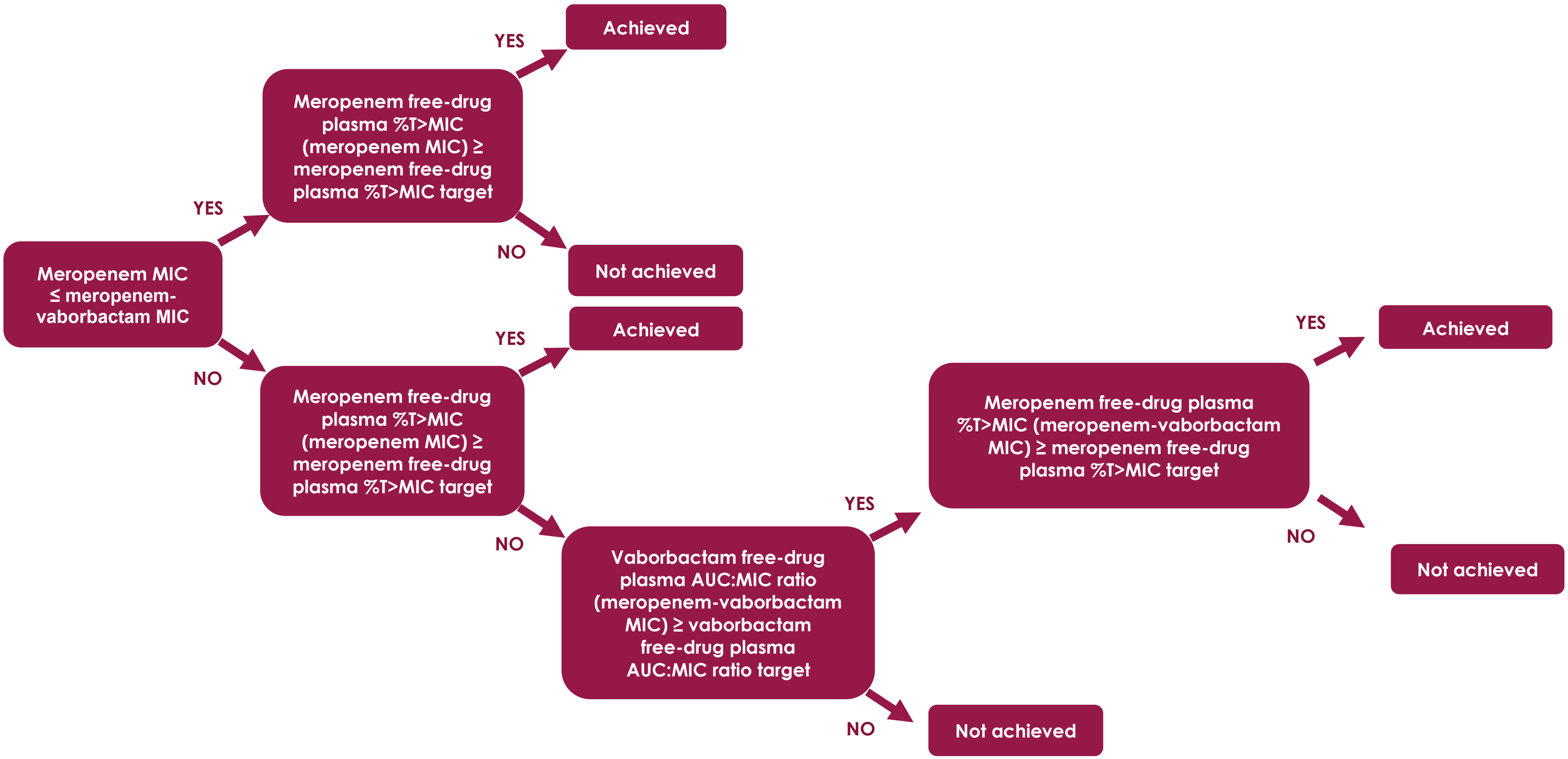

References

1. Castanheira M, Rhomberg PR, Flamm RK, Jones RN. 2016. Effect of the β-lactamase inhibitor vaborbactam combined with meropenem against serine carbapenemase-producing *Enterobacteriaceae*. Antimicrob Agents Chemother 60:5454–5458. https://doi.org/10.1128/AAC.00711-16.

2. Castanheira M, Huband MD, Mendes RE, Flamm RK. 2017.Meropenem-vaborbactam tested against contemporary Gram-negative isolates collected worldwide during 2014, including carbapenem-resistant, KPC-producing, multidrug-resistant, and extensively drug-resistant *Enterobacteriaceae*. Antimicrob Agents Chemother 61:e00567-17. https://doi.org/10.1128/AAC.00567-17.

3. Hackel MA, Lomovskaya O, Dudley MN, Karlowsky JA, Sahm DF. 2018. *In vitro* activity of meropenem-vaborbactam against clinical isolates of KPC-positive *Enterobacteriaceae*. Antimicrob Agents Chemother 62:e01904-17. https://doi.org/10.1128/AAC.01904-17.

4. Pfaller MA, Huband MD, Mendes RE, Flamm RK, Castanheira M. 2018. *In vitro* activity of meropenem/vaborbactam and characterisation of carbapenem resistance mechanisms among carbapenem-resistant *Enterobacteriaceae* from the 2015 meropenem/vaborbactam surveillance programme. Int J Antimicrob Agents 52:144–150. https://doi.org/10.1016/j.ijantimicag.2018.02.021.
